# An Adverse Drug Reaction to Trimethoprim-Sulfamethoxazole Revealing Primary HIV: A Case Report and Literature Review

**DOI:** 10.1155/2015/691010

**Published:** 2015-12-21

**Authors:** Charles Meyer, Nicole Behm, Emily Brown, Nathanial K. Copeland, Marvin J. Sklar

**Affiliations:** ^1^Uniformed Services University of the Health Sciences, Bethesda, MD 20814, USA; ^2^Walter Reed National Military Medical Center, Bethesda, MD 20889, USA; ^3^Naval Medical Research Center, Silver Spring, MD 20910, USA

## Abstract

Adverse drug reactions (ADRs) to antibiotics complicate the management of any infection, particularly opportunistic infections in advanced HIV as some ADRs are potentiated by HIV. Trimethoprim-sulfamethoxazole (TMP-SMX) causes ADRs in 40–80% of HIV infected individuals, compared to 3–5% in the general population. The incidence and severity of ADRs among HIV infected individuals appear to increase as they progress from latent infection to AIDS. We present a single case report of a 55-year-old African American male found to have an otherwise asymptomatic acute HIV infection who developed an ADR to TMP-SMX, despite having previously tolerating the medication. The proposed mechanisms for the increased incidence of sulfa hypersensitivity reactions among HIV infected individuals focus on either (1) HIV-induced changes in the immune function driven by falling levels of CD4 cells or (2) other HIV-specific factors correlated with rising viral load. To our knowledge this is the first reported case of new sulfa hypersensitivity in primary HIV and may provide clinical evidence to support the correlation between viral load and ADRs to TMP-SMX without a severely diminished CD4 count, though further research is necessary. This case also demonstrates a rare and easily overlooked presentation of HIV that may aid in early diagnosis.

## 1. Introduction

Adverse drug reactions (ADRs) to antibiotics complicate the management of any infection, particularly opportunistic infections in advanced HIV as some ADRs are potentiated by HIV [1]. These adverse reactions were previously identified as a possible, initial presentation of acute HIV as early as 1991, but few cases exist within the literature [2]. These reactions may have a varied presentation, ranging from typical hypersensitivity reactions to more severe, although also more rare, manifestations including Stevens-Johnson syndrome, toxic epidermal necrosis, neutropenia, and acute organ failure [1, 3]. Trimethoprim-sulfamethoxazole (TMP-SMX) causes ADRs in 40–80% of HIV infected individuals compared to 3–5% in noninfected individuals [2]. These reactions can cause significant morbidity as TMP-SMX is frequently used to manage opportunistic infections in advanced HIV [4].

The incidence and severity of ADRs among HIV infected individuals appear to increase as they progress from latent infection to AIDS [1]. During the latent phase, HIV infected patients appear to have the same rate of ADRs as noninfected persons [1, 5]. However as the CD4 count declines and viral load increases, the risk of ADRs also increases, with most reactions appearing in patients with CD4 counts less than 100 cells/*μ*L [1, 5]. The proposed mechanisms of the increased sensitivity to ADRs suggest that they may either be immune-related, driven by the falling levels of CD4 cells, or infection-related, driven by accumulation of HIV-specific factors [1, 5, 6]. Clinical correlation to distinguish between these mechanisms is lacking as they occur simultaneously during advanced disease. We present the case of an ADR to TMP-SMX associated with a previously asymptomatic primary HIV infection. To our knowledge this is the first report of such a case, giving a new dimension to what initially appeared to be a straightforward drug reaction, and providing clinical support favoring viral proteins themselves as a significant mechanism in HIV-related sulfa reactions.

## 2. Case Presentation

The patient is an otherwise healthy 55-year-old man who presented with acute onset rash and difficulty breathing three days after incision and drainage and TMP-SMX therapy for a gluteal fold abscess. He denied previous sexually transmitted infections, IV drug use, sexual relations with men, or high-risk sexual behavior. Two days after initiating TMP-SMX, the patient developed a progressive, intensely pruritic rash spreading from his trunk and covering most of his body, including his face, but sparing his palms and soles. On the day of admission, the patient awoke to find his lips swollen and adherent, resulting in difficulty breathing. His initial exam was significant only for mild respiratory distress, severely swollen and tender tongue and lips, and diffuse perioral cracking with fissures. The exam was negative for fever or lymphadenitis. The rash was a diffuse maculopapular rash with no signs of desquamation, infection, or trauma. He was treated with supplemental oxygen, intramuscular epinephrine, and methylprednisolone for suspected anaphylaxis. His labs were significant for moderate elevation in alanine aminotransferase and aspartate aminotransferase and mild leukopenia.

His rash evolved during hospitalization to include his left buccal mucosa and the base of the penile shaft. The oral lesion was a collection of three small, white, painless ulcers on an erythematous base. The penile lesions were two, 1-2 cm painless ulcers with smooth borders and 2-3 cm of surrounding erythema. Given these new findings, he was evaluated for potential infectious etiologies (Table 1). He was found to have a positive HIV viral load at a level consistent with an acute HIV infection, which was subsequently confirmed with a 3rd-generation test 2 weeks later. He would later admit to having unprotected sex with two women he met at a gentlemen's club 15 days prior to initial presentation. He continued to improve and was discharged five days following admission.

## 3. Discussion

TMP-SMX is a commonly used medication that is effective against a wide range of infections. Although the drug is typically well tolerated, HIV infected persons have a particularly high rate of ADRs that significantly impact the management of opportunistic infections in advanced HIV disease [1, 3, 5]. The hypersensitivity to TMP-SMX has been shown to be almost exclusively due to the active metabolites of sulfamethoxazole [1, 6–8]. Sulfamethoxazole is metabolized in the liver to sulfamethoxazole-hydroxylamine, followed by nonenzymatic oxidation to produce nitrososulfamethoxazole (n-SMX) [8]. While it has been shown that n-SMX is toxic to cells in vitro, the exact mechanism remains unclear [8]. What is clear, however, is that under normal physiologic conditions this metabolite is reduced without developing clinically significant toxicity [5, 8]. Yet when there is an imbalance of cellular bioactivation and detoxification mechanisms favoring bioactivation, such as through the depletion of glutathione (GSH) concentrations, hypersensitivity and immune suppression can develop [8].

Additional research has demonstrated that CD4+ and CD19+ cells have relatively greater concentrations of GSH and appear to be less susceptible to the toxic effects of n-SMX than CD8+ cells [6, 7, 9, 10]. This differential sensitivity has been proposed as one mechanism for the increased sensitivity to TMP-SMX in HIV infected individuals: as the HIV infection progresses to AIDS, the relative depletion of CD4+ cells yields CD8+ cell predominance and an overall diminished reduction capacity [5–7]. This diminished capacity favors the bioactivation of SMX to n-SMX over its deactivation and was thought to potentially explain the increased sensitivity to TMP-SMX [8]. However, further research showed that this effect alone could not fully account for the increased incidence and severity of the reactions seen in HIV patients [1]. Rather, it was suggested that there might be an additional, HIV-specific factor contributing to the increased sensitivity [1].

One such factor may be related to an HIV-specific regulatory protein, transactivator of transcription (Tat). Tat is a 14-15 kDa transcription elongation factor that is essential for HIV replication and disease progression [1, 7]. Tat has also been shown to affect the redox status of various cell types, including CD4+ cells, by decreasing the cellular sulfhydryl content, reduced-GSH levels, and GSH synthetase function, ultimately leading to less GSH production [1, 11]. There is also evidence that infected cells secrete Tat which is taken up by noninfected cells where it induces similar changes to their oxidation status [1, 9]. It is suggested that this process could escalate the progression of the disease by decreasing the noninfected cells' ability to withstand oxidative stress [1, 6, 9, 10].

While the correlation between increasing risk of ADRs and progression of HIV infection is well established, it has been difficult to distinguish between the two leading proposals [1, 6–8]. If Tat expression, which is related to viral load, is a significant factor, then ADRs should be increased not only in the late phases of HIV infection when CD4 counts are low, but also during primary HIV when viral loads are increased without a corresponding decrease in CD4 counts. To our knowledge, this is the first case that reports an ADR to TMP-SMX during the primary phase of infection.

Our patient developed a classic hypersensitivity ADR within 72 hours of taking TMP-SMX, a medication he previously tolerated. His symptoms then rapidly resolved following the cessation of the medication. His CD4 count was only drawn once prior to initiating multidrug therapy just over 3 weeks later and was only slightly depressed at 407 cell/*μ*L. While this is well above the typical threshold of less than 100 cells/*μ*L seen in ADRs to TMP-SMX, his viral load was markedly elevated at 9,645,190 copies/*μ*L. Primary HIV can present with similar cutaneous and mucosal findings even in the absence of an ADR; however, it is typically accompanied by fever and lymphadenitis and presents in the first week of illness, none of which were seen in this patient [12]. There are several limitations of this case report, in addition to those inherent in drawing conclusions from a single case. These limitations include the absence of a more extensive laboratory work-up to rule out other infectious causes of the patient's presentation (e.g., EBV) and the lack of additional measurements of CD4 cell counts and HIV viral loads to ensure the discussed conclusions are not based on spurious lab results.

## 4. Conclusion

This patient presented with a suspected severe drug reaction, but further investigation during the evolution of that reaction revealed acute HIV disease and provided potential clinical insight into how HIV may potentiate an adverse reaction to TMP-SMX. Additionally, this case emphasizes how ADRs to sulfa drugs can be a rare initial presentation of acute HIV that might be overlooked in an otherwise healthy individual. While it is not possible to show causality within the limitations of a single case report, this patient's presentation provides clinical evidence to support the correlation between viral load and ADRs to TMP-SMX without a severely diminished CD4 count. Such a correlation would indirectly support the importance of HIV Tat in sensitizing lymphocytes to n-SMX toxicity, rather than a purely immunogenic mechanism dependent on the depletion of CD4+ cells and impaired immune system functioning. Further research is necessary to determine the full role of HIV viral load and Tat in the development of ADRs to TMP-SMX.

## Figures and Tables

**Table 1:** Results of the patient's infectious work-up demonstrating a significantly elevated viral load and slightly depressed CD4 cell count. The remainder of the evaluation was negative, showing no signs of other active infectious processes. Ab, antibody; Ag, antigen; DFA, direct florescent antibody test; CMV, cytomegalovirus; HAV, hepatitis A virus; HBV, hepatitis B virus; HCV, hepatitis C virus; HSV, herpes simplex virus; IgG, immunoglobulin G; IgM, immunoglobulin M; RPR, rapid plasma reagin; VZV, varicella zoster virus.

Lab	Result	Reference range
HIV-1 RNA viral load	9,645,190	Not detected
CD4+	407	441–2156 cell/*µ*L
CD4+/CD8+	0.62	1.0–3.6
HSV Ag by DFA (penis)	(−)	(−)
HSV Ag by DFA (buccal)	(−)	(−)
VZV Ag by DFA	(−)	(−)
HSV 1 Ab IgG	0.91	0.0–0.90
HSV 1 Ab IgM	<1 : 10	<1 : 10
HSV 2 Ab IgG	3.28	0.0–0.90
HSV 2 Ab IgM	<1 : 10	<1 : 10
CMV	(−)	(−)
RPR	(−)	(−)
HAV Ab	(−)	(−)
HBV surface Ag	(−)	(−)
Anti-HBV surface Ab	(+)	(−)
Anti-HBV core Ab IgG	(+)	(−)
Anti-HBV core Ab IgM	(−)	(−)
HCV Ab	(−)	(−)
Parvovirus B19 Ab IgG	4.7	0.0–0.8
Parvovirus B19 Ab IgM	0.3	0.0–0.8
